# Editorial: State of the art body composition profiling: Advances in imaging modalities and patient outcomes

**DOI:** 10.3389/fonc.2022.1096671

**Published:** 2022-12-05

**Authors:** Francesco Petrella, Lucia Manganaro, Stefania Rizzo

**Affiliations:** ^1^ Department of Thoracic Surgery, Istituto di Ricovero e Cura a Carattere Scientifico (IRCCS) European Institute of Oncology, Milan, Italy; ^2^ Department of Oncology and Hemato-oncology, University of Milan, Milan, Italy; ^3^ Department of Radiological, Oncological and Pathological Sciences, University of Rome Sapienza, Rome, Italy; ^4^ Istituto di Imaging della Svizzera Italiana (IIMSI), Ente Ospedaliero Cantonale (EOC), Lugano, Switzerland; ^5^ Facoltà di Scienze Biomediche, Università della Svizzera italiana (USI), Lugano, Switzerland

**Keywords:** body composition, oncology, outcome, sarcopenia, fat

In the last years, body composition (BC) analysis has emerged as a ground-breaking tool that can provide helpful data about nutritional status, in addition to more conventional indicators, as albumin value and body mass index. Recent data disclosed that distinct patterns of BC are associated with different outcomes, in particular in oncologic patients (1).

Anomalies of skeletal muscle – frequently found in oncologic patients - are related to poor outcomes with higher morbidity and mortality rates. Moreover, the distribution and radiodensity of subcutaneous and visceral adipose tissue emerged as determinant prognostic factor for oncologic outcome. Nevertheless, BC assessment is not yet a standard tool of the routine workup of oncologic patients. This is likely due to the heterogeneity of available data, to the shortage of prospective studies and systematic reviews, to the absence of standardized assessment methods, and to the variation of BC among different populations (2).

Hence, we proposed this research topic, on one side, to expand knowledge about the imaging techniques to evaluate BC assessment, ranging from the most conventional ones to the most advanced (including artificial intelligence-aided techniques) (3–5); on the other, to assess the effects of BC on different outcome indicators, as post-operative or chemotherapy-related complications and survival.

In our research topic, we published eight papers: 3 focused on liver diseases, 1 on pancreatic cancer, 1 on ovarian cancer, 1 on lung cancer, 1 on breast cancer and 1 on radiotherapy, thus highlighting the wide variety of fields in which the clinical role of BC is under evaluation.

Del Grande et al. demonstrated that in ovarian cancer patients, computed tomography (CT)-derived body composition profiling might predict the risk of chemotoxicity. In particular, visceral adipose tissue (VAT) and skeletal muscle density (SMD) are associated with chemotherapy cycle delays, whereas skeletal muscle area (SMA) with early discontinuation of chemotherapy.

In daily clinical practice, body surface area (BSA) has been using for chemotherapy dosing, because it is easy to calculate and it discloses an association with fat mass (6). However, BSA often fails to find differences in quantity and proportion of skeletal muscle and fat tissue and so the new proposed indicators (VAT,SMA and SMD) may represent an added value for adequate chemotherapy dosing in ovarian cancer patients.

Rizzo et al. reported that body composition assessment obtained from routine preoperative CT scan may help in predicting postoperative complications following pneumonectomy, with male and female patients showing significant difference in quantity and distribution of fat and muscle tissues. They observed a much higher post-pneumonectomy complication rate in male patients, with the increase of age and the reduction of skeletal muscle area

Considering how severe a complication could be after an extensive pulmonary resection – in particular in case of post resectional bronchopleural fistula (7, 8), the results from this paper may help to optimize the indications and enrolment process, in particular in case of male patients which are more frequently affected by lung cancer.

Fan et al. explored a non-invasive radiomics model based on magnetic resonance imaging (MRI) to recognize the expression of vascular endothelial growth factor (VEGF) in hepatocellular carcinoma before operation. They observed that the combined model acquired both from portal venous and hepatobiliary phase of gadolinium-ethoxybenzyl-diethylenetriamine-pentaacetic acid (Gd-EOB-DTPA)-enhanced MRI can be considered as a reliable prognostic marker for the level of VEGF in hepatocellular carcinoma.

Considering that Anti-VEGF therapy plays a pivotal role in the therapeutic approach to HCC and that several studies disclosed that higher VEGF levels are associated with more aggressive disease (9), preoperative VEGF expression assessment can be crucial for the appropriate therapurtic and prognostic approach.

Cioni et al. proposed a new multi-parametric steatoscore in oncologic patients with non-alcoholic fatty liver disease (NAFLD). They reported high reproducibility and a good correlation with unenhanced CT in evaluation of oncologic patients with NAFLD.

Given the high impact of this disease, diagnosis and quantification of hepatic fat infiltration plays a pivotal role in clinical practice. The gold standard for assessment of fatty liver is liver biopsy (10), but it is an invasive method; therefore, an effective non-invasive method represents an intriguing and promising alternative to more aggressive approach.

Medici et al. provided an overview of the literature about sarcopenia in patients treated with radiotherapy (RT). They confirmed – on the one hand - the adverse influence of sarcopenia in patients suffering from head and neck tumors undergoing RT and, on the other, the chance of enhancing muscle mass and function through nutritional and physical strategies.

The role of sarcopenia in patients receiving RT was first analysed by Dalal et al. which reported 63% incidence of sarcopenia in RT-treated patients with locally advanced pancreatic tumors, highlighting a clear correlation between sarcopenia and outcome (11). Subsequently, other studies disclosed significant correlations between sarcopenia and several clinical outcomes, such as acute toxicity during chemo-radiotherapy in esophageal tumors (12) and survival in patients treated by RT for cervical (13) and head and neck cancers (14).

Rizzo et al. systematically reviewed the association between quantitative body composition values obtained from imaging examinations and chemotherapy-related toxicity in patients suffering from pancreatic cancer (PC). They observed a broad variability of results about the correlation of body composition with chemotherapy-related toxicity in PC patients. In addition, they found that cut-off values to correctly define sarcopenia in PC patients are not yet homogeneously described.

Kripa et al. examined the correlation between body composition values and prognosis in patients affected by metastatic ER+/HER2- breast cancer, receiving therapy with cyclin-dependent kinase (CDK) 4/6 inhibitors. They disclosed a correlation between sarcopenia and progression of disease, disclosing that VAT can positively affect the response to targeted therapy with CDK 4/6 inhibitors.

Bernardi et al. reviewed the literature about the impact of imaging-based values of BC on the outcome of patients receiving liver surgery for hepatocellular carcinoma, cholangiocarcinoma and colorectal liver metastases.

In conclusion, BC assessment relying on imaging-based quantification of muscle mass and fat distribution, is a potential valuable tool to assess the functional status of cancer patients at diagnosis, during treatments and during follow-up.

## Author contributions

All authors contributed to manuscript revision, read, and approved the submitted version.

## References

[1] Radu P Ebadi M Montano-Loz AJ. Dufour JF what is the role of body composition assessment in HCC management? Cancers (Basel) (2022) 14(21):5290. doi: 10.3390/cancers14215290 36358709 PMC9656561

[2] Seo JY Han YM Chung SJ Lim SH Bae JH Chung GE.. Visceral obesity is a more important factor for colorectal adenomas than skeletal muscle or body fat. Cancers (Basel). (2022) 14(21):5256. doi: 10.3390/cancers14215256 36358673 PMC9653975

[3] de Jong EEC van Elmpt W Rizzo S Colarieti A Spitaleri G Leijenaar RTH. Applicability of a prognostic CT-based radiomic signature model trained on stage I-III non-small cell lung cancer in stage IV non-small cell lung cancer. Lung Cancer. (2018) 124:6–11. doi: 10.1016/j.lungcan.2018.07.023 30268481

[4] Manganaro L Lakhman Y Bharwani N Gui B Gigli S Vinci V. Staging, recurrence and follow-up of uterine cervical cancer using MRI: Updated guidelines of the European society of urogenital radiology after revised FIGO staging 2018. Eur Radiol (202) 31(10):7802–16. doi: 10.1007/s00330-020-07632-9 33852049

[5] Bellomi M Rizzo S Travaini LL Bazzi L Trifirò G Zampino MG. Role of multidetector CT and FDG-PET/CT in the diagnosis of local and distant recurrence of resected rectal cancer. Radiol Med (2007) 112(5):681–90. 10.1007/s11547-007-0172-2 17657420

[6] Hunter RJ Navo MA Thaker PH Bodurka DC Wolf JK Smith JA. Dosing chemotherapy in obese patients: Actual versus assigned body surface area (BSA). Cancer Treat Rev (2009) 35(1):69–78. doi: 10.1016/j.ctrv.2008.07.005 18922643

[7] Mazzella A Pardolesi A Maisonneuve P Petrella F Galetta D Gasparri R. Bronchopleural fistula after pneumonectomy: Risk factors and management, focusing on open-window thoracostomy. Semin Thorac Cardiovasc Surg (2018) 30(1):104–13. doi: 10.1053/j.semtcvs.2017.10.003 29109057

[8] Petrella F Borri A Casiraghi M Cavaliere S Donghi S Galetta D. Spaggiari l operative rigid bronchoscopy: indications, basic techniques and results. Multimed Man Cardiothorac Surg (2014) 2014:mmu006. doi: 10.1093/mmcts/mmu006 25133397

[9] Finn RS Zhu AX. Targeting angiogenesis in hepatocellular carcinoma: Focus on VEGF and bevacizumab. Expert Rev Anticancer Ther (2009) 9:503–9. doi: 10.1586/era.09.6 19374603

[10] Bravo AA Sheth SG Chopra S. Liver biopsy. N Engl J Med (2001) 344:495–500. doi: 10.1056/NEJM200102153440706 11172192

[11] Dalal S Hui D Bidaut L Lem K Del Fabbro E Crane C. Relationships among body mass index, longitudinal body composition alterations, and survival in patients with locally advanced pancreatic cancer receiving chemoradiation: A pilot study. J Pain Symptom Manag (2012) 44:181–91. doi: 10.1016/j.jpainsymman.2011.09.010 PMC3990439 22695045

[12] Murimwa GZ Venkat PS Jin W Leuthold S Latifi K Almhanna K. Impact of sarcopenia on outcomes of locally advanced esophageal cancer patients treated with neoadjuvant chemoradiation followed by surgery. J Gastrointest Oncol (2017) 8:808–15. doi: 10.21037/jgo.2017.06.11 PMC5674260 29184684

[13] Kiyotoki T Nakamura K Haraga J Omichi C Ida N Saijo M. Sarcopenia is an important prognostic factor in patients with cervical cancer undergoing concurrent chemoradiotherapy. Int J Gynecol Cancer (2018) 28:168–75. doi: 10.1097/IGC.0000000000001127 29040185

[14] Nishikawa D Hanai N Suzuki H Koide Y Beppu S Hasegawa Y. The impact of skeletal muscle depletion on head and neck squamous cell carcinoma. ORL J Otorhinolaryngol Relat Spec (2018) 80:1–9. doi: 10.1159/000485515 29393251

